# Dietary Essential Amino Acid Intake Is Associated with High Muscle Strength in Korean Older Adults

**DOI:** 10.3390/nu14153104

**Published:** 2022-07-28

**Authors:** Jihyun Im, Hyoungsu Park, Kyong Park

**Affiliations:** 1 Department of Food and Nutrition, Yeungnam University, Gyeongsan 38541, Korea; jhim@ynu.ac.kr; 2 R&D Unit, Maeil Health Nutrition Co., Ltd., Pyeongtaek 17714, Korea; parkhs@maeil.com

**Keywords:** essential amino acid, muscle strength, dietary intake, food source, older adults

## Abstract

The relationship between daily dietary intake of an individual or all essential amino acids (EAAs) and muscle strength in older adults is still inadequately characterized. This population-based cross-sectional study included 5971 participants aged ≥65 years from the 2014–2019 Korea National Health and Nutrition Examination Survey. Dietary information was derived from the 24 h recall data. Total essential amino acid score (EAAS) was calculated with an intake that satisfied the recommended nutrient intake (RNI) in each essential amino acid (EAA). The mean handgrip strength was estimated from triplicate measurements obtained using the dominant hand, and high muscle strength was defined as handgrip strength ≥28 kg for men and ≥18 kg for women. Multivariable-adjusted odds ratios (ORs) and 95% confidence intervals (CIs) were estimated using logistic regression models. After multivariable adjustment, we found that a high total EAAS was associated with high muscle strength in Korean older adults (OR: 1.38, 95% CI: 1.07–1.79). High muscle strength was significantly enhanced with increased total EAA intake from animal sources (OR: 1.27, 95% CI: 1.02–1.58), but there was no significant association with total EAA intake from non-animal sources. EAA intake and high muscle strength are associated based on a positive dose-response relationship in which high muscle strength is further increased when the overall EAA intake meets the RNI. Thus, Korean older adults should ensure an adequate intake of all EAAs from various food sources (especially animal sources) to meet the RNI as a prerequisite for achieving high muscle strength.

## 1. Introduction

In 2020, the proportion of older adults (≥65 years) had reached 15.7% of the total population in Korea, which is projected to become a superaged society in 2025, with the proportion of older adults rising to 20.3% [1]. Population aging requires an increased focus on geriatric diseases. For example, muscle loss and weakening during older adulthood are associated with an increased prevalence of sarcopenia and other comorbidities [2], leading to a diminished quality of life [3], indicating an urgent need for measures to address these medical conditions.

Age-related muscle weakening can be reduced by adequate protein intake [4]. However, in Korea, the protein intake is much lower in older adults aged ≥65 years than in other age groups; specifically, the number of people with inadequate animal protein intake is rising [5]. The proportions of people not meeting the estimated average requirement for protein are 30.7% among men aged 65–74 years, 35.6% among women aged 65–74 years, 48.0% among men aged ≥75 years, and 60.1% among women aged ≥75 years, indicating that approximately half of the elderly population in Korea is at high risk for protein deficiency [6].

The essential amino acids (EAAs), which cannot be synthesized by the body and must come from the diet, have a direct effect on muscle protein synthesis in the body [7,8]. Several recent clinical trials that examined the effects of essential amino acid (EAA) supplements on muscle strength and muscle mass have reported beneficial health outcomes [9,10,11]. According to a randomized 12-week intervention study on 38 healthy older adults aged 65–80 years in Italy, the EAA supplement group showed a significant increase in muscle strength and appendicular lean mass [9]. In a US study that examined the association between the daily intake of 15 g of EAA supplements for 24 weeks and muscle strength in 45 older adults aged ≥65 years, the EAA supplements increased EAA-stimulated muscle protein synthesis, and those participants who performed physical activity in addition to taking EAA supplements showed a significant increase in muscle strength [10]. An 8-week randomized intervention study conducted on 44 patients with sarcopenia in Japan reported that the intake of leucine-enriched amino acid supplements was associated with significantly increased handgrip strength [11]. Although many clinical trials have indicated that EAAs have a positive effect on muscle mass and strength [9,10,11], these studies generally assessed the effects of short-term use of supplements in a small sample, whereas large-scale community-based studies focused on the health impact of the daily dietary intake of EAAs are lacking. A few epidemiological studies were conducted on the Japanese population, reporting a sex-specific association of EAA with lean mass and frailty in the elderly [12,13]. However, it remains unclear whether the daily dietary intake of individual or all EAAs is sufficient to meet the recommended nutrient intake (RNI) and whether intake levels of EAAs are associated with muscle strength in the Korean elderly population at high risk of protein deficiency. The objective of this study was to use the data from the 2014–2019 Korea National Health and Nutrition Examination Survey (KNHANES) for calculating a score that evaluates the achieved intake levels relative to the Dietary Reference Intakes for Koreans (KDRIs) and for assessing the association between the EAA score (EAAS) and high muscle strength among Korean older adults aged ≥65 years.

## 2. Materials and Methods

### 2.1. Study Population

The KNHANES is a large-scale cross-sectional survey launched in 1998 that provides nationally representative and reliable statistics pertaining to the public health status, health behaviors, and food and nutrient intake and can be used as baseline data for health policies [14]. The KNHANES data were collected by trained staff in accordance with standardized guidelines, and the survey was divided into health interview, health examination, and nutritional survey [14]. In the present study, we analyzed data from a six-year period (2014–2019) that included a parameter for grip strength as the main outcome measure in this study.

From a total of 47,309 participants of the 2014–2019 KNHANES, the following participants were excluded from our analysis: aged <65 years (*n* = 37,484), those with missing values for grip strength (*n* = 1476), those diagnosed with cancer or severe cerebrovascular and cardiovascular disease at the time of the survey (*n* = 1710), and those with daily total energy intake <500 kcal or >5000 kcal (*n* = 668). Thus, a total of 5971 participants were included in the analysis.

The data were collected after obtaining written informed consent from all participants. The survey was approved by the Institutional Review Board (IRB) at the Korea Disease Control and Prevention Agency (IRB numbers: 2013-12EXP-03-5C, 2018-01-03-P-A, 2018-01-03-C-A). For the years 2015–2017, the survey was exempted for IRB review as research for public welfare [14].

### 2.2. Demographic and Lifestyle Information

Sex, age, household income, smoking status, alcohol consumption, and physical activity were obtained through the health questionnaire survey in mobile examination buses [14]. The monthly average equivalent household income was calculated considering age and sex, and participants were classified as low, mid-low, mid-high, and high household income groups. Smoking was assessed based on current smoking status. Individuals with responses “smoke every day” and “smoke occasionally” were considered current smokers, and those with responses “smoked in the past but not anymore” or “don’t smoke” were considered nonsmokers. Drinking was surveyed as the drinking frequency during the past year in the raw data, which were converted to weekly drinking frequency and multiplied by the amount of alcohol consumed per sitting. Participants who consumed alcohol were considered drinkers, and those who did not consume alcohol were considered non-drinkers. Physical activity was assessed using the vigorous/moderate physical activity and walking parameters that were divided into work and leisure. The number of days of each physical activity (vigorous/moderate/walking) was multiplied by time to compute the duration of weekly physical activity, and this value was weighted for the exercise intensity. The resulting metabolic equivalents (METs-hour/week) were divided into tertiles (low, mid, high). The use of dietary supplements was surveyed through in-person interviews for the nutritional survey [14], and responses were divided into yes or no for the “use of dietary supplements for at least two weeks in the past year.”

### 2.3. Dietary Amino Acid and Total EAAS Calculation

Nutrient intake was surveyed by a trained interviewer through an in-person interview using the 24 h recall method [14]. Supplementary materials were used to improve participants’ recall [14]. In this study, the daily dietary intake of EAAs was derived from the 24 h recall data obtained from the 2014–2019 KNHANES.

The EAA levels in food were calculated using our previously established amino acid database [15], based on the Food Composition Table ver. 9.2 (Rural Development Administration, Jeonju, Korea) and the Computer Aided Nutritional Analysis Program 4.0 (The Korean Nutrition Society, Seoul, Korea). The details of the development of the amino acid database can be found in the study by Chae et al., (2020) [15].

The intake values were calculated for the nine EAAs (leucine, isoleucine, valine, lysine, histidine, threonine, methionine, phenylalanine, and tryptophan). Furthermore, for each EAA, the achieved intake level was scored for every participant based on the sex- and age-specific RNI values according to the 2020 KDRIs [6]. A score of 1 was given for meeting the RNI values for individual EAAs, and a total EAAS ranging from 0–9 was derived from the scores for satisfying the RNI values for all nine EAAs. The computed total EAAS values were divided into quartiles for the analysis.

Dietary sources of EAAs included animal sources (meat and meat products, fish and shellfish, eggs, and milk and dairy products) and non-animal sources (grains and grain products, potatoes and starch, sugars, legumes and legume products, nuts and seeds, vegetables, mushrooms, fruits, seaweeds, fat and oils, beverages, seasoning, and cooked/processed foods).

### 2.4. Definition of High Muscle Strength

Hand grip strength measurements were performed with all participants aged ≥10 years during the health examinations since the KNHANES 2014 [16]. The hand grip strength is the force applied for manipulating an object by coordinating four fingers and the thumb, which was measured using a digital grip strength dynamometer (T.K.K 5401, Takei Scientific Instruments Co.,Ltd., Niigata City, Japan) [14]. For participants with a single dominant hand, we used the average value of three grip strength measurements with the dominant hand. For participants claiming to use both hands equally, we used the average value of grip strength measurements performed three times with the right and the left hand. High muscle strength was defined by a hand grip strength ≥28 kg for men and ≥18 kg for women in accordance with the Asian Working Group for Sarcopenia 2019 [17].

### 2.5. Statistical Analysis

We analyzed the 2014–2019 KNHANES data using a complex sample design that considered stratified variables, cluster variables, and weights. The participants’ general characteristics according to the total EAAS are presented as frequency values (%) using the chi-square test for categorical variables or the mean ± standard error using linear regression for continuous variables. The association between the total EAAS and high muscle strength was evaluated using the odds ratio (OR) and 95% confidence interval (CI) obtained by multivariable logistic regression analysis. Potential confounders used in the analyses were determined based on the preliminary analysis and previous studies that examined the association between amino acid intake and grip strength [13,18,19]. We checked for effect modifiers (e.g., demographic, lifestyle, or dietary factors) using the multiplicative term in the statistical model, but there was no effect modifier that could alter the association between the total EAAS and high muscle strength. We built following statistical models: Model 1, unadjusted model; Model 2, adjusted for age, sex, smoking status, alcohol consumption, and household income; Model 3, further adjusted for body mass index (BMI), physical activity, use of dietary supplements, and total energy intake. *p* for trend was computed applying the quartiles median values for the total EAAS. To test for nonlinearity between the total EAAS and high muscle strength, restricted cubic splines were performed. Three knots were used for the analysis, and adjustments were made for the same covariates used in Model 3. All statistical analyses were performed using the Statistical Analysis System (SAS) ver. 9.4 (SAS Institute, Cary, NC, USA), and the statistical significance level for all tests was set at α = 0.05.

## 3. Results

We summarized the general characteristics of 5971 participants (≥65 years) of the 2014–2019 KNHANES based on the total EAAS quartiles (Table 1). The total EAAS medians were 5.62 for quartile 1, 7.61 for quartile 2, 8.32 for quartile 3, and 8.99 for quartile 4. Higher levels of total EAAS were associated with a higher percentage of men (*p* < 0.001), an older age (*p* < 0.001), and a higher percentage of the highest household income quartile (*p* < 0.001). Furthermore, the percentage of those who consumed alcohol higher with total EAAS (*p* < 0.001), and nonsmokers (*p* < 0.001) and people who engage in a high level of physical activity tended to have a high total EAAS (*p* < 0.001). In contrast, there were no significant differences in BMI across the total EAAS quartiles (*p* = 0.12). Higher levels of total EAAS were associated with a higher intake of total energy, carbohydrate, fat, protein, and protein/body weight (all, *p* < 0.001). The percentage of people who use dietary supplements was approximately 17% higher in quartile 4 of total EAAS than quartile 1 (*p* < 0.001). Higher levels of total EAAS were associated with a higher handgrip strength (*p* < 0.001).

We analyzed the association between total EAAS quartiles and high muscle strength (Table 2). In the unadjusted model, the OR for high muscle strength significantly increased with increasing total EAAS (Model 1, *p* for trend <0.001). Specifically, the OR for high muscle strength was 2.17 times higher in the group with the highest total EAAS (Q4) than in the group with the lowest total EAAS (Model 1, OR: 2.17, 95% CI: 1.83–2.57). In the model adjusted for age, sex, smoking status, alcohol consumption, household income, BMI, physical activity, use of dietary supplements, and total energy intake (Model 3), this association was weakened, but there was still a significant linear association (*p* for trend = 0.005). The OR for high muscle strength was 1.38 times higher in the group with the highest total EAAS (Q4) than in the group with the lowest total EAAS (OR: 1.38, 95% CI: 1.07–1.79).

The intake levels of individual EAAs were presented as the percentage of the RNI for the total EAAS (Table 3). In this analysis, the intake values for the nine EAAs increased with increasing total EAAS (all *p* < 0.001). Although the participants with high total EAAS values (Q3 and Q4) typically met the RNIs for most individual EAAs, participants in Q3 showed a low intake of lysine (89.22%), methionine (74.74%), and phenylalanine (71.63%) relative to the RNI. The percentages of RNI were very low for participants with the lowest total EAAS (Q1) compared with those values for the participants in Q3 and Q4. In the Q1 group, the percentages of the RNIs for lysine, methionine, and phenylalanine were especially low at 33.81% ± 0.84%, 30.92% ± 0.63%, and 34.64% ± 0.45%, respectively.

We also assessed the association between total EAA intake and high muscle strength based on the type of source for the EAAs (animal or non-animal sources, see Table 4). In the unadjusted Model 1, an increase in the intake of total EAAs from animal sources was associated with significantly increased OR values for high muscle strength (*p* for trend <0.001). Specifically, in the group with the highest total EAA intake from animal sources (Q4), the OR value for high muscle strength was 2.16 times higher than in the group with the lowest total EAA intake from animal sources (OR: 2.16, 95% CI: 1.81–2.59). In Model 2, which was based on data adjusted for age, sex, smoking status, alcohol consumption, and household income, the participants in the group with the highest total EAA intake from animal sources (Q4) were 39% more likely to have high muscle strength than the participants in the group with the lowest total EAA intake from animal sources (Q1) (OR: 1.39, 95% CI: 1.14–1.69). In Model 3, which was adjusted for all covariates, this association was weakened but still significant; the group with the highest total EAA intake from animal sources had a 1.27 times higher OR for high muscle strength than the group with the lowest total EAA intake from animal sources (OR: 1.27, 95% CI: 1.02–1.58), and there was a linear association between total EAA intake and high muscle strength (*p* for trend = 0.046). An assessment of total EAA intake from non-animal sources showed that the group with the highest total EAA intake from non-animal sources had a 2.16-times higher OR for high muscle strength than the group with the lowest total EAA intake from non-animal sources (Model 1, OR: 2.16, 95% CI: 1.82–2.56). In Model 2, which was adjusted for age, sex, smoking status, alcohol consumption, and household income, this association was weakened but still displayed significant linearity (*p* for trend <0.001). Specifically, the group with the highest total EAA intake from non-animal sources (Q4) had a 1.46-times higher OR for high muscle strength than the group with the lowest total EAA intake from non-animal sources (OR: 1.46, 95% CI: 1.20–1.77). However, there was no significant association between the total EAA intake from non-animal sources and high muscle strength in Model 3 that was adjusted for age, sex, smoking status, alcohol consumption, household income, BMI, physical activity, use of dietary supplements, and total energy intake (OR: 1.18, 95% CI: 0.91–1.54, *p* for trend = 0.34).

**Figure 1:**
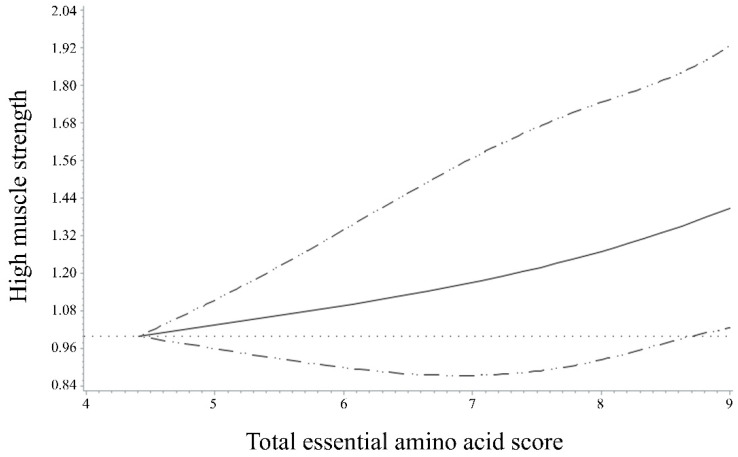
Odds ratios (95% confidence intervals) for the nonlinear relationship between total essential amino acid score and high muscle strength in Korean older adults were evaluated with restricted cubic splines. The model was adjusted for age, sex, smoking status, alcohol consumption, household income, physical activity, body mass index, dietary supplement use, and total energy intake. *p* for nonlinearity = 0.74.

Figure 1 shows the spline curves that represent the nonlinear association between total EAAS and high muscle strength. The data were adjusted for covariates, including sex, age, smoking status, alcohol consumption, household income, BMI, physical activity, use of dietary supplements, and total energy intake. There was positive association between the total EAAS and high muscle strength. We found that increasing total EAAS values were associated with increasing ORs for high muscle strength in a dose-response relationship (*p* for nonlinearity = 0.74).

## 4. Discussion

In this study, we evaluated the level of meeting the RNI of dietary EAAs and analyzed the association between total EAAS and high muscle strength among older adults aged ≥65 years in Korea using the 2014–2019 KNHANES data. High muscle strength was significantly increased when the RNI for all EAAs was met, and there was a positive dose–response relationship between the total EAAS and high muscle strength. We further found that an increase in the intake of total EAAs from animal sources was associated with a significant increase in high muscle strength, but we did not detect a significant association between high muscle strength and total EAA intake from non-animal sources.

We also observed that an increase in total EAAS was associated with a significantly increased OR for high muscle strength, which corroborated similar findings reported previously [12,13]. A positive correlation between dietary EAA intake and lean body mass (r = 0.71, *p* = 0.02) was reported in a study that analyzed the correlation between dietary protein, amino acid intake, and lean body mass in healthy older men [12]. A cross-sectional association between dietary amino acid intake and frailty was identified in a study conducted on women aged ≥65 years in Japan, in which the prevalence of frailty was reduced by 31% in the group with the highest EAA intake compared with that in the group with the lowest EAA intake [13]. Maintaining and increasing the muscle mass requires the dietary intake of adequate amounts of proteins that meet the RNI [20,21] as opposed to simply increasing the overall protein intake [22]. The RNI is the minimum amount of a nutrient needed to reduce the risk of chronic disease, and failure to meet the RNI may increase the risk of malnutrition and various other diseases [23]. In a study that analyzed the association between protein intake adequacy against the RNI and low skeletal muscle index in adults aged ≥50 years using the 2008–2011 KNHANES data, the authors reported that the group with a protein intake not meeting the RNI had a significantly higher prevalence of low skeletal muscle index than the group meeting the RNI for proteins in both men and women (men, OR: 1.59, 95% CI: 1.22–2.07; women, OR: 1.39, 95% CI: 1.12–1.72) [21]. Older adults have weaker stimulation of protein synthesis [20], and particularly, Korean older adults show inadequate protein intake relative to the RNI [21,24]. Therefore, the daily intake of sufficient amounts of dietary protein to ensure an adequate supply of EAAs is important for stimulating protein synthesis, and thus maintaining muscle strength and skeletal muscle mass [20,21,25].

Muscles are a major target of dietary protein, and they are more affected by changes in protein intake than other tissues [22]. Especially the EAAs, which are not synthesized in the body, are known to stimulate muscle synthesis [7,8,26,27,28] by activating the mammalian/mechanistic target of rapamycin complex 1 pathway [29]. Many studies have attempted to investigate the association between specific EAAs and muscle strength [30,31,32]. However, supplementing only a specific amino acid may induce absorption competition between amino acids that results in an imbalance of amino acids [33]. Therefore, it is important to consume adequate amounts of all EAAs to promote intracellular protein synthesis [34,35]. In this study, we observed that the participants who met the RNI for all EAAs through balanced meals had a higher muscle strength. Specifically, our results highlighted the importance of EAA intake from animal sources. Meat (beef, pork, chicken), fish, eggs, and milk and dairy products are well-characterized sources of proteins that contain all EAAs [6,36]. However, plant proteins often lack one or more EAAs [6], and they are less effective in inducing muscle anabolism than animal proteins due to their low digestion-absorption rates [37]. In relation to this fact, an earlier study indicated the importance of branched-chain amino acids from non-grain food products and various other food sources for older adults aged ≥65 years in Korea. This earlier study demonstrated that handgrip strength is negatively associated with the intake of branched-chain amino acids from grains (β-coefficient = −0.641, *p* for trend = 0.03), whereas it is significantly positively associated with the intake of branched-chain amino acids from non-grain food sources (β-coefficient = 0.832, *p* for trend = 0.02), which stresses the need to consume proteins from various food sources other than grains [30]. However, the majority of Korean adults aged ≥65 years rely heavily on grains or plant food products for protein [38,39,40], with an extremely low intake of high-quality animal proteins [5,6]. Hence, it is important for older adults to consume an adequate amount of high-quality protein during meals to ensure an adequate supply of EAAs [4,20,41,42]. Moreover, consuming a balanced amount of all EAAs that meet the RNI should be emphasized.

In this study, we examined the intake level of each EAA in the Korean older adult population, and we observed that this population has a particularly low intake of phenylalanine, methionine, and lysine. Phenylalanine is generally obtained from eggs, chicken, liver, beef, and milk [6,43], and it is converted to tyrosine and plays a role in the synthesis of neurotransmitters [44]. Methionine is found in eggs, cheese, and chicken [45], and is used in the synthesis of an antioxidant known as glutathione [46]. Lysine is a limiting amino acid in rice but is highly abundant in beef and poultry [6], and it is involved in muscle remodeling wherein a dynamic equilibrium is maintained through continuous protein synthesis and degradation; a deficiency of lysine may induce growth delay and anemia [6,43,47]. In summary, phenylalanine, methionine, and lysine, which are not adequately consumed by Korean older adults, can be generally obtained from animal food sources. However, Korean older adults primarily consume plant proteins instead of animal proteins based on their prolonged high-carbohydrate and plant-based diet [40]. A study that examined the current status of dietary EAA intake by food group in the Korean population observed that histidine, isoleucine, leucine, valine, and tryptophan are mostly consumed from grains and grain products [15]. Although most EAAs can be obtained from animal sources, Korean older adults enjoy a high-carbohydrate and plant-based diet and based on consuming high amounts of grains, which appears to have contributed to the supplementation of several EAAs from grains [15]. However, food from animal sources has high levels of phenylalanine, methionine, and lysine compared with levels of other EAAs [43], which could have contributed to the inadequate intake of these EAAs among Korean older adults who do not consume an adequate amount of food from animal products. Therefore, we emphasize the need to obtain high quality of proteins from animal sources rather than from plant sources.

This study has a few limitations. First, although we performed a stepwise adjustment of confounders based on a literature review and preliminary analysis when analyzing the association between EAA intake and grip strength, there is still the possibility of a residual confounding effect. Second, the KNHANES is a cross-sectional study that collected information about EAA intake and grip strength at the same time point. Thus, we cannot establish a reverse causality bias between the two factors. Hence, it remains unclear whether EAA intake contributed to the enhanced grip strength or whether people with high grip strength consumed high amounts of EAAs. Third, the participants’ diets were assessed based on a single 24 h recall data survey; thus, it is possible that this data set does not accurately reflect the usual diet. In addition, supplementary intakes of EAAs were not considered due to the lack of this information in KNHANES. However, trained interviewers collected the study data using standardized guidelines, and we attempted to minimize this limitation by excluding participants with extreme daily total energy intake (<500 kcal or >5000 kcal). Fourth, we cannot eliminate the possibility of underestimation of amino acid intake because some amino acid contents in foods were not provided when developing the database although we included more food items that had different processing or preparation methods (e.g., dried vs. fresh) by applying the moisture conversion factor.

This study is significant because the total EAASs were generated in the context of the Korean society using the sex- and age-specific RNIs for amino acids presented in the 2020 KDRI. Furthermore, there is a lack of sound Korean databases; however, here, we established a dietary amino acid database that is linked to large-scale epidemiology data, and we assessed the status of EAA intake among Korean older adults, all of which will provide guidance for addressing these nutritional shortcomings.

## 5. Conclusions

In conclusion, we assessed the achieved intake levels relative to the RNIs for dietary EAAs among Korean older adults aged ≥65 years, and we observed that the intake of phenylalanine, methionine, and lysine failed to meet the RNI in this population. Furthermore, we observed a positive dose-response relationship between total EAA intake and high muscle strength, in which high muscle strength increases when the overall EAA intake meets the RNI. Thus, to ensure high muscle strength, Korean older adults need to consume an adequate amount of EAAs from various food sources (especially food from animal sources) that meet the RNI. Amid a lack of studies analyzing the association between dietary EAAs and high muscle strength, this study presents critical data pertaining to EAA intake and appropriate food sources to promote high muscle strength. Hence, prospective cohort studies that longitudinally examine the association between dietary EAAs and high muscle strength should be conducted along with large clinical trials to establish causality of this relationship.

## Figures and Tables

**Table 1:** General characteristics of the participants according to total EAAS, KNHANES 2014–2019 (*n* = 5971).

	Total EAAS				*p* ^1^
	Q1	Q2	Q3	Q4	
	*n* = 1492	*n* = 1493	*n* = 1493	*n* = 1493	
Score, median (range)	5.62 (0.83–6.89)	7.61 (6.90–7.96)	8.32 (7.96–8.70)	8.99 (8.70–9.00)	
Age (years)	73.90 ± 0.13	73.10 ± 0.13	72.35 ± 0.13	71.62 ± 0.13	<0.001
Sex					<0.001
Men	585 (39.21)	627 (42.00)	684 (45.81)	674 (45.14)	
Women	907 (60.79)	866 (58.00)	809 (54.19)	819 (54.86)	
Household income					<0.001
Low	889 (59.99)	750 (50.51)	661 (44.48)	509 (34.35)	
Mid-low	345 (23.28)	440 (29.63)	424 (28.53)	433 (29.22)	
Mid-high	169 (11.40)	163 (10.98)	237 (15.95)	304 (20.51)	
High	79 (5.33)	132 (8.89)	164 (11.04)	236 (15.92)	
Alcohol consumption					<0.001
Drinkers	592 (41.96)	746 (51.59)	810 (55.75)	851 (58.49)	
Non-drinkers	819 (58.04)	700 (48.41)	643 (44.25)	604 (41.51)	
Smoking status					0.04
Smokers	160 (11.35)	135 (9.37)	129 (8.90)	121 (8.32)	
Nonsmokers	1250 (88.65)	1306 (90.63)	1321 (91.10)	1333 (91.68)	
Body mass index (kg/m^2^)	23.95 ± 0.082	24.12 ± 0.082	23.92 ± 0.082	24.21 ± 0.082	0.12
Physical activity ^2^					<0.001
Low	516 (39.30)	445 (32.58)	421 (30.46)	329 (23.45)	
Mid	451 (34.35)	479 (35.07)	479 (34.66)	523 (37.28)	
High	346 (26.35)	442 (32.36)	482 (34.88)	551 (39.27)	
Dietary intake					
Total energy (kcal/day)	1124.11 ± 13.46	1499.31 ± 13.46	1747.98 ± 13.46	2286.87 ± 13.46	<0.001
Carbohydrate (g/day)	213.85 ± 2.62	276.05 ± 2.61	304.54 ± 2.61	363.90 ± 2.61	<0.001
Fat (g/day)	11.79 ± 0.43	19.00 ± 0.43	28.21 ± 0.43	46.57 ± 0.43	<0.001
Protein (g/day)	30.90 ± 0.51	44.96 ± 0.51	56.96 ± 0.51	85.37 ± 0.51	<0.001
Protein/body weight (g/kg)	0.54 ± 0.01	0.76 ± 0.01	0.96 ± 0.01	1.41 ± 0.01	<0.001
Individual EAAS ^3^					
Leucine	0.66 ± 0.002	0.96 ± 0.002	0.99 ± 0.002	0.98 ± 0.003	<0.001
Isoleucine	0.75 ± 0.003	1.00 ± 0.002	0.99 ± 0.002	0.98 ± 0.003	<0.001
Valine	0.79 ± 0.003	1.00 ± 0.002	0.99 ± 0.002	0.98 ± 0.003	<0.001
Lysine	0.34 ± 0.003	0.59 ± 0.002	0.87 ± 0.002	1.00 ± 0.003	<0.001
Histidine	0.65 ± 0.003	0.96 ± 0.002	0.99 ± 0.002	0.98 ± 0.003	<0.001
Threonine	0.71 ± 0.003	0.99 ± 0.002	0.99 ± 0.002	0.98 ± 0.003	<0.001
Methionine	0.32 ± 0.002	0.51 ± 0.002	0.74 ± 0.002	0.97 ± 0.003	<0.001
Phenylalanine	0.39 ± 0.002	0.56 ± 0.002	0.71 ± 0.002	0.90 ± 0.002	<0.001
Tryptophan	0.89 ± 0.002	1.00 ± 0.002	0.99 ± 0.002	0.98 ± 0.003	<0.001
Dietary supplement use					<0.001
Yes	602 (40.35)	740 (49.56)	804 (53.89)	859 (57.54)	
No	890 (59.65)	753 (50.44)	688 (46.11)	634 (42.46)	
Handgrip strength (kg)	21.87 ± 0.22	23.35 ± 0.22	24.66 ± 0.22	25.07 ± 0.22	<0.001

EAAS, Essential Amino Acid Score; KNHANES, Korea National Health and Nutrition Examination Survey; Q, Quartile. Values are mean ± standard error or N (%). ^1^ *p* values were derived from χ^2^ test for categorical variables, and *p* for trends across the quartile of EAAS were calculated using linear regression models for continuous variables. ^2^ Physical activity was categorized into 3 groups, according to tertiles of metabolic equivalents (METs)-hours/week. ^3^ Values were adjusted for sex, age, and total energy intake.

**Table 2:** Multivariable logistic regression analysis between total EAAS and high muscle strength, KNHANES 2014–2019 (*n* = 5971).

	Total EAAS				*p* for Trend
	Q1	Q2	Q3	Q4	
	*n* = 1492	*n* = 1493	*n* = 1493	*n* = 1493	
Score, median	5.62	7.61	8.32	8.99	
Case (%)	735 (49.26)	861 (57.67)	944 (63.23)	1014 (67.92)	
Model 1	1	1.34 (1.13–1.60)	1.80 (1.52–2.13)	2.17 (1.83–2.57)	<0.001
Model 2	1	1.15 (0.95–1.39)	1.37 (1.14–1.64)	1.57 (1.30–1.90)	<0.001
Model 3	1	1.14 (0.93–1.40)	1.36 (1.10–1.69)	1.38 (1.07–1.79)	0.005

EAAS, Essential Amino Acid Score; KNHANES, Korea National Health and Nutrition Examination Survey; Q, Quartile. Model 1: unadjusted. Model 2: adjusted for age, sex, smoking status, alcohol consumption, and household income. Model 3: further adjusted for body mass index, physical activity, dietary supplement use, and total energy intake.

**Table 3:** Individual EAA intake as a percentage of the RNI for Koreans according to total EAAS.

	Total EAAS				*p*
	Q1	Q2	Q3	Q4	
	*n* = 1492	*n* = 1493	*n* = 1493	*n* = 1493	
RNI (%)					
Leucine	63.79 ± 0.90	102.09 ± 0.90	137.73 ± 0.90	220.35 ± 0.90	<0.001
Isoleucine	73.48 ± 1.13	119.85 ± 1.13	164.83 ± 1.13	269.25 ± 1.13	<0.001
Valine	78.50 ± 1.03	123.10 ± 1.03	164.12 ± 1.03	255.77 ± 1.03	<0.001
Lysine	33.81 ± 0.84	58.84 ± 0.84	89.22 ± 0.84	157.74 ± 0.84	<0.001
Histidine	64.13 ± 1.19	105.14 ± 1.19	146.57 ± 1.19	246.01 ± 1.19	<0.001
Threonine	69.68 ± 1.11	112.98 ± 1.11	156.96 ± 1.11	257.11 ± 1.11	<0.001
Methionine	30.92 ± 0.63	51.37 ± 0.63	74.74 ± 0.63	128.54 ± 0.63	<0.001
Phenylalanine	34.64 ± 0.45	54.51 ± 0.45	71.63 ± 0.45	111.60 ± 0.45	<0.001
Tryptophan	99.30 ± 1.78	154.69 ± 1.78	204.29 ± 1.78	322.84 ± 1.78	<0.001

EAA, Essential Amino Acid; RNI, Recommended Nutrient Intake; EAAS, Essential Amino Acid Score; Q, Quartile. Values are mean ± standard error.

**Table 4:** Multivariable logistic regression analysis between total EAA intake and high muscle strength according to food from animal and non-animal source, KNHANES 2014–2019 (*n* = 5971).

Food Group	Total EAA Intake (g)				*p* for Trend
	Q1	Q2	Q3	Q4	
Animal source ^1^					
*n*	1426	1426	1427	1426	
Case (%)	730 (21.13)	846 (24.49)	891 (25.79)	988 (28.60)	
Intake, median (g/day)	0.4	3.3	6.8	14.2	
Model 1	1	1.34 (1.13–1.58)	1.53 (1.29–1.82)	2.16 (1.81–2.59)	<0.001
Model 2	1	1.13 (0.93–1.38)	1.10 (0.91–1.32)	1.39 (1.14–1.69)	0.002
Model 3	1	1.12 (0.91–1.37)	1.07 (0.88–1.30)	1.27 (1.02–1.58)	0.046
Non-animal source ^2^					
*n*	1492	1493	1493	1493	
Case (%)	738 (20.77)	877 (24.68)	915 (25.75)	1024 (28.81)	
Intake, median (g/day)	6.0	8.8	11.7	17.0	
Model 1	1	1.29 (1.10–1.52)	1.57 (1.32–1.88)	2.16 (1.82–2.56)	<0.001
Model 2	1	1.17 (0.97–1.40)	1.14 (0.93–1.40)	1.46 (1.20–1.77)	<0.001
Model 3	1	1.13 (0.93–1.39)	1.03 (0.82–1.30)	1.18 (0.91–1.54)	0.34

EAA, Essential Amino Acid; KNHANES, Korea National Health and Nutrition Examination Survey; Q, Quartile. Model 1: unadjusted. Model 2: adjusted for age, sex, smoking status, alcohol consumption, and household income. Model 3: additionally adjusted for body mass index, physical activity, dietary supplement use, and total energy intake. ^1^ Animal source includes meat and meat products, fish and shellfish, milk and dairy products, and eggs. ^2^ Non-animal source includes grains and grain products, potatoes and starch products, sugar and sugar products, legumes and legume products, nuts and seeds, vegetables, mushrooms, fruits, seaweeds, oils, beverages, seasoning, and cooked/processed foods, and other products.
